# Mathematical modelling of infrared‐dried kiwifruit slices under natural and forced convection

**DOI:** 10.1002/fsn3.1212

**Published:** 2019-09-26

**Authors:** Ebrahim Sadeghi, Ali Haghighi Asl, Kamyar Movagharnejad

**Affiliations:** ^1^ Faculty of Chemical, Petroleum and Gas Engineering Semnan University Semnan Iran; ^2^ Faculty of Chemical Engineering Babol Noshirvani University of Technology Babol Iran

**Keywords:** diffusivity, infrared dryer, kiwifruit, thin‐layer mathematical modeling

## Abstract

In this work, the effect of the radiation intensity, slice thickness, and the distance between slices and infrared lamps under natural drying air and the effect of slice thickness and air velocity under forced drying air on the moisture diffusion characteristics and the drying rate of kiwifruit slices during infrared drying were investigated. The drying of kiwifruit happened in the falling rate period, and no constant‐rate period was observed in the drying curves. One hundred models were fitted to the drying data. Among the models, the exponential dsecay function model and modified two‐term exponential‐V model and the artificial neural networks with 4‐5‐7‐1 and 3‐5‐5‐1 topologies, hyperbolic tangent sigmoid transfer function, and Levenberg‐Marquardt training algorithm presented the best results and showed the goodness of fit with the experimental data for the former and latter systems, respectively. The diffusivities varied between 1.216 × 10^−10^–8.997 × 10^−10^ m^2^⁄s and 2.567 × 10^−10^–10.335 × 10^−10^ m^2^⁄s for natural and forced drying air systems, respectively.

## 1 INTRODUCTION

Drying is one of the oldest moisture removal method, which has always been a great way of preserving foods by human beings (Ertekin & Firat, 2017). Infrared (IR) heating has been surveyed either alone or in combination with the other drying methods for food and agricultural materials with different moisture contents such as rough rice (Abe & Afzal, 1997), potato (Afzal & Abe, 1998), red pepper (Nasiroglu & Kocabiyik, 2009), and peach (Wang & Sheng, 2006). This is due to its many attributes such as the simplicity of the required equipment, shortened drying time and more uniform heating along with improved product quality, and lower airflow through the product. It is particularly emphasized in the literature that IR drying method is valid for products with considerable moisture content, owing to the fact that water molecules almost totally absorb wavelengths >3 μm (Celma, Rojas, & Lopez‐Rodriguez, 2008).

The mathematical modelling of the drying process and the equipment are the most relevant aspects of the drying technology (Toğrul, 2006). It is widely applied to foresee drying behavior of materials being dried, design new dryers, and control of the process (Beigi, Torki‐Harchegani, & Mahmoodi‐Eshkaftaki, 2016).

Having a thin‐layer drying equation representing moisture exchange between a thin layer of the drying product with its surrounding air is fundamental to the drying simulation (Wang, Fon, Fang, & Sokhansanj, 2004). Because of the appearance of one or more parameters in such models, the parameters can be found as a function of the drying conditions (Jurendić, 2012). Despite numerous studies on mathematical modelling of drying, no theoretical model was found that is practical and can unify the calculations and the observed progress has largely limited to experimental ones (Erbay & Icier, 2010).

Thin‐layer drying equations involve theoretical, semitheoretical, and empirical models. The former based on the conceptions of the fundamental phenomena (Beigi et al., 2016). It has been proved that semitheoretical and empirical models are only useful and practical when designing dryers (Ertekin & Firat, 2017).

In general, food drying happens under a falling rate period and during which diffusion is considered as the most likely physical mechanism governing the moisture movement (Das, Das, & Bal, 2009). Fick's second law of diffusion takes into account the dependence of the transport attributes on temperature, moisture content, and pressure. Assuming no temperature gradient within the product and the negligible effect of pressure on most drying processes, its Lumped model emerges. The values of effective moisture diffusivity predicted by analytical solution of the Lumped model for an infinite slab (Equation 1) were in close proximity to the experimental values (da Silva, Precker, & de Lima, 2009).

(1) $$\text{MR}=\frac{8}{\pi^{2}}\sum_{n=0}^{\infty }\frac{1}{{\left(2n+1\right)}^{2}}\exp\left[-\frac{{\left(2n+1\right)}^{2}\pi^{2}D_{\text{eff}}t}{L^{2}}\right]$$

where, MR is the dimensionless moisture ratio, *L* is the thickness of the slice if drying occurs from only one side (m), *t* is time (s).

The effective moisture diffusivity, *D*_eff_ (m^2^/s), is assumed as representative of all mechanisms influencing the mass transfer phenomenon, which illustrates the moisture movement toward the outside (Corrêa, de Oliveira, Baptestini, Diniz, & da Paixão, 2012).

Equation 1 can be reduced for sufficiently long drying times (MR < 0.6) as follows (Doymaz, 2014b):

(2) $$\text{MR}=\frac{8}{\pi^{2}}\exp\left(-\frac{\pi^{2}D_{\text{eff}}t}{L^{2}}\right)$$

The method of slopes is employed in the estimation of effective moisture diffusivity of samples at corresponding moisture contents under different drying conditions (Çağlar, Toğrul, & Toğrul, 2009).

(3) $$\ln\text{MR}=\ln\left(\frac{8}{\pi^{2}}\right)-\frac{\pi^{2}D_{\text{eff}}}{L^{2}}t$$

Equation 3 indicates that the change of ln (MR) values versus *t* is linear. After determining the slope, the *D*_eff_ can be calculated easily by replacing the values of the slice thickness in Equation 3.

Moisture diffusivity and activation energy are fundamental to design an appropriate dryer of food and agricultural products (Chayjan, Kaveh, & Khayati, 2014). Some authors studied the significant effect of temperature on *D*_eff_ and presented an Arrhenius type exponential relationship to describe it (Celma et al., 2008; Darvishi, Najafi, Hosainpour, Khodaei, & Aazdbakht, 2013):

(4) $$D_{\text{eff}}=D_{0}\exp\left(-\frac{E_{a}}{\text{RT}}\right)$$

where, *D*_0_, *E*_a_, *R*, and *T* are the reference diffusion coefficient at infinitely high temperature (m^2^/s), the activation energy for diffusion (J/mol), the universal gas constant (J/mol.*K*), and the drying chamber temperature (*K*), respectively.

Increase in diffusivity with reduction in moisture content is assigned to higher product temperature in the final stage of drying process, which leads to maximum effective diffusivity at the end of drying process (Das et al., 2009).

When it is not possible to measure the quantity of temperature in the radiation power level during the drying process, a product mass and a power level‐dependent Arrhenius type diffusivity are used to calculate the activation energy in different drying systems (Doymaz, 2015a):

(5) $$D_{\text{eff}}=D_{1}\exp\left(-\frac{E_{a}m}{P}\right)$$

where *D*_1_, *m*, *P*, and *E*_a_ are the pre‐exponential factor, the weight of the raw material (g), the infrared output power (W), and the activation energy for the drying of the product (W/g).

If the determination coefficient cannot be high enough, other factors affecting *D*_eff_ have to be considered. The most appropriate method in this situation is reflecting these factors to *D*_eff_ and performing nonlinear regression analysis to fit the data (Jurendić, 2012). The values of *D*_eff_ for food systems are mostly in order of 10^−8^ to 10^−12^ m^2^/s (Doymaz, 2015b).

In addition, in recent years, numerous authors have applied the artificial neural networks (ANNs) modelling methods for simulation of drying processes. Jurendić (2012) found the ANN with 4‐9‐9‐1 topology trained with LM algorithm and LOGSIG transfer function as the most suitable model to forecast response values. Topologies of 3‐2‐3‐1 and 3‐3‐3‐1 with the LM training algorithm and transfer functions of TANSIG, TANSIG‐LOGSIG ‐TANSIG as the best structures were suggested by Chayjan et al. (2014) for the prediction of effective diffusivity and energy consumption at sour cherry drying process, respectively.

Although a large number of thin‐layer mathematical models have widely been applied to describe the drying process, a very little information is available for moisture diffusivity of kiwifruit under infrared drying conditions. However, there is little‐to‐no information available about the effect of infrared power (IP), slice thickness (*λ*), slices distance from the IR lamps (∆), and air velocity (*V*) on drying behavior of kiwifruit slices under natural and forced drying air systems in the literature according to the authors' knowledge.

The main goals of this investigation were to study the effect of the aforementioned parameters on the drying kinetics; to find the best model to describe infrared radiation drying; and to compute the effective moisture diffusivity and activation energy of kiwifruit slices. Note that these approaches are crucial for the design and the setting‐up of dryers for particular sample products.

## 2 MATERIALS AND METHODS

### 2.1 Materials

Kiwifruits were prepared from a local market in Amol, Iran. In order to decelerate the respiration, physiological, and chemical changes (Mohammadi, Rafiee, Keyhani, & Emam‐Djomeh, 2009), all samples were stored in a refrigerator at 4 ± 0.5°C for at least 48 hr prior to the drying process. The samples were placed outside of the refrigerator for approximately 1 hr to reach room temperature and then peeled and sliced into 2, 4, and 6 mm thick and about 40 mm diameter. The initial moisture content found to be around 4.7 g water/g dry solid (d.b.). The drying tests were carried out down to a final moisture content of about 0.20 d.b. similar to that reported for kiwifruit (Diamante, Durand, Savage, & Vanhanen, 2010).

### 2.2 The experimental equipment and procedures

Thin‐layer drying of kiwifruit slices was done in a laboratory‐scale single tray IR dryer that was designed and made at the Babol Noshirvani University of Technology, Iran. Airflow can easily enter into drying chamber through holes at the bottom of the dryer and leave by natural convection through some holes provided on the two opposing walls.

To leave airflow as forced convection, the drying chamber is replaced with a similar one equipped with a variable speed fan located on one of the walls. The slices were uniformly distributed on the tray inside the drying chamber. To obtain the drying curves, moisture loss was continuously recorded by using a digital electronic balance of ±0.1 g accuracy (EK‐6100i series, A&D Company). Thin‐layer drying experiments of kiwifruit slices were done at three levels of radiation intensity, 1,000, 1,500, and 2,000 W, three levels of slice thickness, 2, 4, and 6 mm, and three levels of distance between slices and infrared lamps, 550, 700, and 850 mm; and three levels of slice thickness, 2, 4, and 6 mm and three levels of air velocity, 1, 1.25, and 1.5 m/s, under natural and forced drying air systems, respectively.

A data acquisition system, which was connected to the computer, was applied to measure the weight loss of the kiwifruit slices at specified time intervals. Prior to the drying experiments in order to ensure steady state in tests and to avoid the drift of the weighting arising from the increase of the air temperature inside the chamber, the IR dryer was left running without any slice for about 80–100 min.

The drying experiments were carried out with respect to a Box–Behnken design and a central composite design formularized by Design Expert 7.0 software (DX7) for natural and forced convection runs, respectively. The changes in moisture ratios of the samples over the drying time obtained from each run under fixed operating parameters were fitted to the mathematical models (Table 1) using the Minitab 18 statistical software. The constants of each model were computed based on Levenberg–Marquardt (LM) algorithm up to 200 iterations with convergence tolerance of 0.000001 and a confidence interval of 95%. These values were then correlated with operating parameters using the DX7 software under the same design. In the same way, the diffusivities were correlated with operating parameters. The constants of mathematical models and the diffusivities were chosen as response variables, and IP, *λ*, ∆, and *V* were selected as the main operating variables depending on the type of experiments underway.

**Table 1:** Mathematical models employed for fitting of infrared radiation experimental data

Model no.	Model name	Model	References
1	Lewis (Newton)	$\text{MR}=\exp\left(-kt\right)$	Sharma et al. (2005a)
2	Page	$\text{MR}=\exp\left(-kt^{n}\right)$	Abe and Afzal (1997)
3	Modified Page ‐I	$\text{MR}=\exp\left[{\left(-kt\right)}^{n}\right]$	Beigi et al. (2016)
4	Modified Page ‐II	$\text{MR}=\exp\left[-{\left(\mathrm{kt}\right)}^{n}\right]$	Celma et al. (2009)
5	Modified Page ‐III	$\text{MR}=\exp\left[-{\left(-kt\right)}^{n}\right]$	Ertekin and Firat (2017)
6	Modified Page ‐IV	$\text{MR}=a\;\exp\left[-{\left(\mathrm{kt}\right)}^{n}\right]$	
7	Modified Page ‐VI	$\text{MR}=\exp\left[kt^{n}\right]$	
8	Modified Page ‐VII	$\text{MR}=\exp\left[-k{\left(t/L^{2}\right)}^{n}\right]$	
9	Modified Page ‐VIII	$\text{MR}=\exp\left\{-{\left[k\left(t/L^{2}\right)\right]}^{n}\right\}$	
10	Modified Page ‐IX	$\text{MR}=k\;\exp\left[{\left(-t/L^{2}\right)}^{n}\right]$	
11	Otsura et al.	$\text{MR}=1-\exp\left[-\left(kt^{n}\right)\right]$	
12	Henderson and Pabis	$\text{MR}=a\;\exp\left(-kt\right)$	Das et al. (2009)
13	Logarithmic (Asymptotic)	$\text{MR}=a\;\exp\left(-kt\right)+c$	Beigi et al. (2016), Corrêa et al. (2012) and Darvishi et al. (2013)
14	Midilli‐Kucuk (Midilli or Midilli et al.)	$\text{MR}=a\;\exp\left(-kt^{n}\right)+b\times t$	Abano et al. (2014) and Corrêa et al. (2012)
15	Modified Midilli‐I	$\text{MR}=\exp\left(-kt^{n}\right)+b\times t$	Doymaz (2014a)
16	Modified Midilli‐II	$\text{MR}=\exp\left(-kt\right)+b\times t$	Ertekin and Firat (2017)
17	Modified Midilli‐III	$\text{MR}=a\;\exp\left(-kt\right)+b\times t$	Doymaz (2014a)
18	Demir et al.	$\text{MR}=a\;\exp\left[{\left(-kt\right)}^{n}\right]+b$	Chayjan et al. (2014)
19	Two‐term exponential	$\text{MR}=a\;\exp\left(-k_{1}t\right)+b\;\exp\left(-k_{2}t\right)$	Celma et al. (2009), Doymaz (2014c) and Erbay and Icier (2010)
20	Modified two‐term exponential ‐I	$\text{MR}=a\;\exp\left(-kt\right)+\left(1-a\right)\;\exp\left(-kat\right)$	
21	Modified two‐term exponential ‐II	$\text{MR}=a\;\exp\left(k_{0}t\right)+\left(1-a\right)\;\exp\left(-k_{1}t\right)$	Ertekin and Firat (2017)
22	Modified two‐term exponential ‐III	$\text{MR}=a\;\exp\left(k_{0}t\right)+\left(1-a\right)\;\exp\left(k_{1}t\right)$	
23	Modified two‐term exponential ‐IV	$\text{MR}=a\;\exp\left(-k_{0}t\right)+a\;\exp\left(-k_{1}t\right)$	
24	Modified two‐term exponential ‐V	$\text{MR}=a\;\exp\left(-k_{0}t^{n}\right)+b\;\exp\left(-k_{1}t\right)$	Doymaz (2014a)
25	Modified two‐term exponential ‐VI (Verma et al.)	$\text{MR}=a\;\exp\left(-k_{0}t\right)+\left(1-a\right)\;\exp\left(-k_{1}t\right)$	Corrêa et al. (2012)
26	Modified Henderson and Pabis ‐I	$\text{MR}=a\;\exp\left(-kt\right)+b\;\exp\left(-gt\right)+c\;\exp\left(-ht\right)$	Celma et al. (2009)
27	Modified Henderson and Pabis ‐II	$\text{MR}=a\;\exp\left(-kt^{n}\right)+b\;\exp\left(-gt\right)+c\;\exp\left(-ht\right)$	Ertekin and Firat (2017)
28	Simplified Fick	$\text{MR}=k\;\exp\left[-c\left(t/L^{2}\right)\right]$	Toğrul (2005)
29	Thompson	$t=a\;\ln\left(\text{MR}\right)+b\;{\left[\ln\left(\text{MR}\right)\right]}^{2}$	Erbay and Icier (2010)
30	Wang and Singh	$\text{MR}=1+at+bt^{2}$	
31	Hii et al.	$\text{MR}=a\;\exp\left(-kt^{n}\right)+c\;\exp\left(-gt^{n}\right)$	Doymaz (2014a)
32	Weibull distribution ‐I	$\text{MR}=a-b\;\exp\left[-kt^{n}\right]$	Ertekin and Firat (2017)
33	Weibull distribution ‐III	$\text{MR}=\exp\left[-{\left(t/a\right)}^{n}\right]$	Doymaz (2015a)
34	Vega‐Galvez et al. ‐I	$\text{MR}=n+k\sqrt{t}$	Ertekin and Firat (2017)
35	Vega‐Galvez et al. ‐II	$\text{MR}=\exp\left(n+kt\right)$	Doymaz (2014c)
36	Vega‐Galvez et al. ‐III	$\text{MR}={\left(a+bt\right)}^{2}$	Doymaz (2015b)
37	Jena Das	$\text{MR}=a\;\exp\left(-kt+b\sqrt{t}\right)+c$	Ertekin and Firat (2017)
38	Wang et al. (one term)	$\text{MR}=a\;\exp\left(\mathrm{bkt}\right)+\left(1-a\right)$	Wang et al. (2004)
39	Wang et al. (two term)	$\text{MR}=\left(1-a\right)\;\exp\left(\mathrm{bkt}\right)+a\;\exp\left(\mathrm{ckt}\right)$	
40	Wang et al. (three term)	$\text{MR}=\left(1-a-b\right)\;\exp\left(\mathrm{ckt}\right)+a\;\exp\left(\mathrm{dkt}\right)+b\;\exp\left(\mathrm{fkt}\right)$	
41	Diamente et al.	$\ln\left[-\ln\left(\text{MR}\right)\right]=a+b\;\ln\left(t\right)+c{\left[\ln\left(t\right)\right]}^{2}$	Ertekin and Firat (2017)
42	Haghi and Angiz ‐I	$\text{MR}=a\;\exp\left(-bt^{n}\right)+dt^{2}+et+f$	
43	Haghi and Angiz ‐II	$\text{MR}=a+bt+ct^{2}+dt^{3}$	
44	Haghi and Angiz ‐III	$\text{MR}=\frac{a+bt}{1+ct+dt^{2}}$	
45	Haghi and Angiz ‐IV	$\text{MR}=a\;\exp\left[-\frac{{\left(t-b\right)}^{2}}{2c^{2}}\right]$	
46	Sripinyowanich and Noomhorm	$\text{MR}=\exp\left(-kt^{n}\right)+bt+c$	
47	Noomhorm and Verma	$\text{MR}=a\;\exp\left(-kt\right)+b\;\exp\left(-gt\right)+c$	
48	Hasibuan and Daud	$\text{MR}=1-at^{n}\;\exp\left(-kt^{m}\right)$	
49	Henderson and Henderson ‐I	$\text{MR}=c\left[\exp\left(-bt\right)+\frac{1}{9}\exp\left(-9kt\right)\right]$	
50	Henderson and Henderson ‐II	$\text{MR}=c\;\exp\left(-bt\right)+\frac{1}{9}\exp\left(-9kt\right)$	
51	Parabolic	$\text{MR}=a+bt+ct^{2}$	Doymaz (2015a)
52	Geometric	$\text{MR}=at^{-n}$	Ertekin and Firat (2017)
53	Logistic	$\text{MR}=\frac{a}{1+b\;\exp\;\left(\mathrm{kt}\right)}$	Chayjan et al. (2014)
54	Power Law	$\text{MR}=at^{b}$	Ertekin and Firat (2017)
55	Regression ‐I	$\text{MR}=\exp\left[-\left(ct^{2}+bt\right)\right]$	
56	Regression ‐II	$t=a{\left(\text{MR}\right)}^{2}+b\left(\text{MR}\right)+c$	
57	Chavez‐Mendez et al.	$\text{MR}=a+b\;\ln\left(t\right)$	
58	Aghbashlo et al.	$\text{MR}=\exp\left[-\frac{k_{1}t}{\left(1+k_{2}t\right)}\right]$	Doymaz et al. (2016)
59	Modified Henderson and Perry	$\text{MR}=a\;\exp\left(-kt^{n}\right)$	Ertekin and Firat (2017)
60	Alibas	$\text{MR}=a\;\exp\left[{\left(-kt\right)}^{n}+bt\right]+g$	
	**Growth curve models**		
61	Baroreflex five‐parameter function (baro 5)	$\text{MR}=c+\frac{d-c}{1+f\;\exp\left\{b_{1}\left[\log\left(t\right)-\log\left(e\right)\right]\right\}+\left(1-f\right)\exp\left\{b_{2}\left[\log\left(t\right)-\log\left(e\right)\right]\right\}}$ $f=\frac{1}{1+\exp\left\{\frac{2b_{1}b_{2}}{\left|b_{1}+b_{2}\right|}\left[\log\left(t\right)-\log\left(e\right)\right]\right\}}$	Ritz Strebig and Ritz (2016 )
62	Brain‐Cousens (BC.4)	$\text{MR}=\frac{d+ft}{1+\exp\left\{b\left[\log\left(t\right)-\log\left(e\right)\right]\right\}}$	
63	Brain‐Cousens (BC.5)	$\text{MR}=c+\frac{d-c+ft}{1+\exp\left\{b\left[\log\left(t\right)-\log\left(e\right)\right]\right\}}$	
64	Four‐parameter Cedergreen‐Ritz‐Streibig function (CRS.4a)	$\begin{array}{cc} \text{UCRS}.4a;\;\alpha =1 & \text{MR}=\frac{d+f\;\exp\left(-1/t^{\alpha }\right)}{1+\exp\left\{b\left[\log\left(t\right)-\log\left(e\right)\right]\right\}} \end{array}$	
65	CRS.4b	CRS.4b;α=0.5	
66	CRS.4c	CRS.4c;α=0.25	
67	Four‐parameter Cedergreen‐Ritz‐Streibig function for describing u‐shaped hormesis (UCRS.4a)	$\begin{array}{cc} \text{UCRS}.4a;\;\alpha =1 & \text{MR}=d-\frac{d+f\;\exp\left(-1/t^{\alpha }\right)}{1+\exp\left\{b\left[\log\left(t\right)-\log\left(e\right)\right]\right\}} \end{array}$	
68	UCRS.4b	UCRS.4b;α=0.5	
69	UCRS.4c	UCRS.4c;α=0.25	
70	Five‐parameter Cedergreen‐Ritz‐Streibig function (CRS.5a)	$\begin{array}{cc} \text{CRS}.5a;\;\alpha =1 & \text{MR}=\frac{d-c+f\;\exp\left(-1/t^{\alpha }\right)}{1+\exp\left\{b\left[\log\left(t\right)-\log\left(e\right)\right]\right\}} \end{array}$	
71	CRS.5b	CRS.5b;α=0.5	
72	CRS.5c	CRS.5c;α=0.25	
73	Five‐parameter Cedergreen‐Ritz‐Streibig function for describing u‐shaped hormesis (UCRS.5a)	$\begin{array}{cc} \text{UCRS}.5a;\;\alpha =1 & \text{MR}=c+d-\frac{d-c+f\;\exp\left(-1/t^{\alpha }\right)}{1+\exp\left\{b\left[\log\left(t\right)-\log\left(e\right)\right]\right\}} \end{array}$	
74	UCRS.5b	UCRS.5b;α=0.5	
75	UCRS.5c	UCRS.5c;α=0.25	
76	Six‐parameter Cedergreen‐Ritz‐Streibig function (CRS.6)	$\begin{array}{cc} \text{CRS}.6 & \text{MR}=c+\frac{d-c+f\;\exp\left(-1/t^{\alpha }\right)}{1+\exp\left\{b\left[\log\left(t\right)-\log\left(e\right)\right]\right\}} \end{array}$	
77	Two‐parameter log‐logistic function (LL.2)	$\text{MR}=\frac{1}{1+\exp\left\{b\left[\log\left(t\right)-\log\left(e\right)\right]\right\}}$	
78		$\text{MR}=\frac{1}{1+\exp\left\{b\left[\log\left(t\right)-e\right]\right\}}$	
79	Three‐parameter log‐logistic function (LL.3)	$\text{MR}=\frac{d}{1+\exp\left\{b\left[\log\left(t\right)-\log\left(e\right)\right]\right\}}$	
80		$\text{MR}=\frac{d}{1+\exp\left\{b\left[\log\left(t\right)-e\right]\right\}}$	
81	Three‐parameter log‐logistic function with the upper limit 1 (LL.3u)	$\text{MR}=c+\frac{1-c}{1+\exp\left\{b\left[\log\left(t\right)-\log\left(e\right)\right]\right\}}$	
82		$\text{MR}=c+\frac{1-c}{1+\exp\left\{b\left[\log\left(t\right)-e\right]\right\}}$	
83	Four‐parameter log‐logistic function (LL.4)	$\text{MR}=c+\frac{d-c}{1+\exp\left\{b\left[\log\left(t\right)-\log\left(e\right)\right]\right\}}$	
84		$\text{MR}=c+\frac{d-c}{1+\exp\left\{b\left[\log\left(t\right)-e\right]\right\}}$	
85	Five‐parameter log‐logistic function (LL.5)	$\text{MR}=c+\frac{d-c}{{\left(1+\exp\left\{b\left[\log\left(t\right)-\log\left(e\right)\right]\right\}\right)}^{f}}$	
86		$\text{MR}=c+\frac{d-c}{{\left(1+\exp\left\{b\left[\log\left(t\right)-e\right]\right\}\right)}^{f}}$	
87	Exponential dacay function (EXD.3)	$\text{MR}=c+\left(d-c\right)\exp\left(-t/e\right)$	
88	Gompertz growth (G.4)	$\text{MR}=c+\left(d-c\right)\left[\exp\left(-\exp\left(b\left(t-e\right)\right)\right)\right]$	
89	Log normal functions	$\begin{array}{cc} \text{LN}.2: & \text{MR}=\frac{1}{t\sigma \sqrt{2\pi }}\exp\left\{-\frac{{\left\{\ln\left(t\right)-\mu \right\}}^{2}}{2\sigma^{2}}\right\} \end{array}$	
90		$\begin{array}{cc} \text{LN}.3: & \text{MR}=\frac{1}{\left(t-\gamma \right)\sigma \sqrt{2\pi }}\exp\left\{-\frac{{\left\{\ln\left(t-\gamma \right)-\mu \right\}}^{2}}{2\sigma^{2}}\right\} \end{array}$	
91		$\begin{array}{cc} \text{LN}\text{.3u}\left(\sigma =1\right) & \text{MR}=\frac{1}{\left(t-\gamma \right)\sqrt{2\pi }}\exp\left\{-\frac{{\left\{\ln\left(t-\gamma \right)-\mu \right\}}^{2}}{2}\right\} \end{array}$	
92		$\begin{array}{cc} \text{LN}.4: & \text{MR}=\frac{1}{\left(\frac{t-\beta }{\alpha -t}\right)\sigma \sqrt{2\pi }}\exp\left\{-\frac{{\left\{\ln\left(\frac{t-\beta }{\alpha -t}\right)-\mu \right\}}^{2}}{2\sigma^{2}}\right\} \end{array}$	
93	Two‐parameter Weibull functions	$\begin{array}{cc} W1.2: & \text{MR}=\exp\left(-\exp\left(b\left(\log\left(t\right)-e\right)\right)\right) \end{array}$	
94		$\begin{array}{cc} W2.2: & \text{MR}\;={\left(\frac{\beta }{\alpha }\right)}^{\beta }{\left(\frac{t}{\alpha }\right)}^{\beta -1}\exp\left(-{\left(\frac{t}{\alpha }\right)}^{\beta }\right) \end{array}$	
95	Three‐parameter Weibull functions	$\begin{array}{cc} W1.3: & \text{MR}=d\;\exp\left(-\exp\left(b\left(\log\left(t\right)-e\right)\right)\right) \end{array}$	
96		$\begin{array}{cc} W2.3: & \text{MR}=\frac{\alpha }{\beta }{\left(\frac{t-\mu }{\beta }\right)}^{\alpha -1}\exp\left(-{\left(\frac{t-\mu }{\beta }\right)}^{\alpha }\right) \end{array}$	
97	Four‐parameter Weibull functions	$\begin{array}{cc} W1.4: & \text{MR}=c+\left(d-c\right)\exp\left(-\exp\left(b\left(\log\left(t\right)-\log\left(e\right)\right)\right)\right) \end{array}$	
98		$\begin{array}{cc} W1.4: & \text{MR}=c+\left(d-c\right)\left(1-\exp\left(-\exp\left(b\left(\log\left(t\right)-\log\left(e\right)\right)\right)\right)\right) \end{array}$	
99		$\begin{array}{cc} W2.4 & \text{MR}=\frac{k}{\vartheta }{\left(\frac{t-\alpha }{2\beta \vartheta }\right)}^{k-1}t^{-1}\exp\left(-{\left(\frac{t-\alpha }{2\beta \vartheta }\right)}^{k}\right) \end{array}$	
100	Feed‐forward neural networks		

### 2.3 Mathematical modeling of drying

Equation 6 shows the moisture content of slices at any time of drying (*M_t_*, (d.b.)) (da Silva et al., 2009):

(6) $$M_{t}=\frac{W_{t}-W_{\text{dm}}}{W_{\text{dm}}}$$

where, *W_t_* and *W*_dm_ are the weight of the kiwifruit slices at any time of drying (g) and their dry solid weight (g), respectively.

Dimensionless moisture ratio (MR), representing the existing moisture content at any time in the kiwifruit slices to the amount of initial moisture, and calculated using Equation 7 (Çağlar et al., 2009; Celma, López‐Rodríguez, & Cuadros, 2009):

(7) $$\text{MR}=\frac{M_{t}-M_{e}}{M_{0}-M_{e}}$$

where, *M*_0_, *M_t_*, and *M*_e_ are the initial moisture content, the mean moisture content at time *t*, and the equilibrium moisture content, respectively. Since at IR process, slices may be dried as much as dry solid content (Celma et al., 2008). Hence, *M*_e_ is relatively small compared to *M_t_* or *M*_0_ and is considered zero. Therefore, MR can be rewritten as in Equation 8 (Darvishi et al., 2013; Shi et al., 2008):

(8) $$\text{MR}=\frac{M_{t}}{M_{0}}$$

The drying rate (DR) of kiwifruit slices, with regard to the change in moisture content in each consecutive time interval, was calculated according to Equation 9 (Doymaz, 2014b, 2015a; Doymaz, Karasu, & Baslar, 2016):

(9) $$\text{DR}=\frac{M_{t}-M_{t+\Delta t}}{\Delta t}$$

where, *M_t_*_+∆_ *_t_* is moisture content at *t*+∆*t*.

In addition, in this study, feed‐forward networks trained with Levenberg‐Marquardt backpropagation algorithm (trainlm), containing sigmoid function in hidden layer(s) and linear output function as transfer functions, were employed to fit experimental data. In this work 70%, 15%, and 15% data were used for training, validation, and testing, respectively. The input layers were composed of four datasets of radiation intensity, slice thickness, distances between slices and infrared lamps, and drying time, and three datasets of slice thickness, air velocity and drying time for natural and forced drying air systems, respectively. ANNs with different neurons in the hidden layer(s) in the range of default 0–1,000 epochs were tested. The optimal number of neurons in the hidden layer(s) was sought by trial and error method and accordingly to statistical analysis.

### 2.4 Effective moisture diffusivity

The *D*_eff_ values are designated by plotting empirical drying data of each run in terms of ln MR against drying time (Equation 3) and following that the *E*_a_ value can be calculated by performing linear regression analysis and plotting ln *D*_eff_ against 1/*T* (Equation 4) and or plotting ln *D*_eff_ against *m*/*P_m_* at a given thickness of slices (Equation 5) depending on the available data. The Equations 4 and 5 are usually provided for a given thickness of the samples in the literature. The equations of *D*_eff_ were presented as Equations 4 and 5 using Excel 2013 and the Minitab 18. To consider other factors affecting *D*_eff_, polynomial equations were presented using the DX7 software under the same design for both systems.

### 2.5 Statistical analysis

Generally, determination coefficient (*R* ^2^), reduced chi‐square (*χ* ^2^) test, and root mean square error (RMSE) analysis were applied as the most common methods for fitting quality of the experimental data to the models in the literature. In addition, modeling efficiency (EF), mean bias error (MBE), and mean relative percentage error (*P*) were utilized as the criteria to choose the best equation to account for all of the changes observed in the drying curves of the dehydrated samples (Beigi et al., 2016; Doymaz, 2014b, 2015b; Doymaz et al., 2016; Ertekin & Firat, 2017).

(10) $$R^{2}=1-\left[\frac{\sum_{i=1}^{N}{\left({\text{MR}}_{\text{pre},i}-{\text{MR}}_{\exp,i}\right)}^{2}}{\sum_{i=1}^{N}{\left({\bar{\mathrm{MR}}}_{\text{pre}}-{\text{MR}}_{\exp,i}\right)}^{2}}\right]$$

(11) $$\chi^{2}=\frac{\sum_{i=1}^{N}{\left({\text{MR}}_{\exp,i}-{\text{MR}}_{\text{pre},i}\right)}^{2}}{N-n}$$

(12) $$\text{RMSE}=\sqrt{\frac{\sum_{i=1}^{N}{\left({\text{MR}}_{\text{pre},i}-{\text{MR}}_{\exp,i}\right)}^{2}}{N}}$$

(13) $$P=\frac{100}{N}\sum_{i=1}^{N}\frac{\left|{\text{MR}}_{\text{pre},i}-{\text{MR}}_{\exp,i}\right|}{{\text{MR}}_{\exp,i}}$$

(14) $$\text{EF}=\frac{\sum_{i=1}^{N}{\left({\text{MR}}_{\exp,i}-{\bar{\text{MR}}}_{\exp}\right)}^{2}-\sum_{i=1}^{N}{\left({\text{MR}}_{\text{pre},\;i}-{\text{MR}}_{\exp,\;i}\right)}^{2}}{\sum_{i=1}^{N}{\left({\text{MR}}_{\exp,\;i}-{\bar{\text{MR}}}_{\exp}\right)}^{2}}$$

(15) $$\text{MBE}=\frac{1}{N}\sum_{i=1}^{N}\left({\text{MR}}_{\text{pre},i}-{\text{MR}}_{\exp,i}\right)$$

MR_exp,_ *_i_*, MR_pre,_ *_i_* are the *i*th experimental and predicted moisture ratios; *N* and *n* are the number of observations in every run and the number of constants in the model, respectively.

The closer the value of *R* ^2^ to 1, the better the agreemt between the empirical and predicted values. The lower values of *χ* ^2^ and RMSE result in the better goodness of the fit (Toğrul, 2005, 2006). The values of *P* <5%, <10%, and >10% show a very high, a good, and a poor goodness of fit for practical purposes, respectively. EF exhibits the fitting strength of the model and the highest value of it is 1. The ideal value of MBE is zero (Ertekin & Firat, 2017).

## 3 RESULT AND DISCUSSION

**Figure 1:**
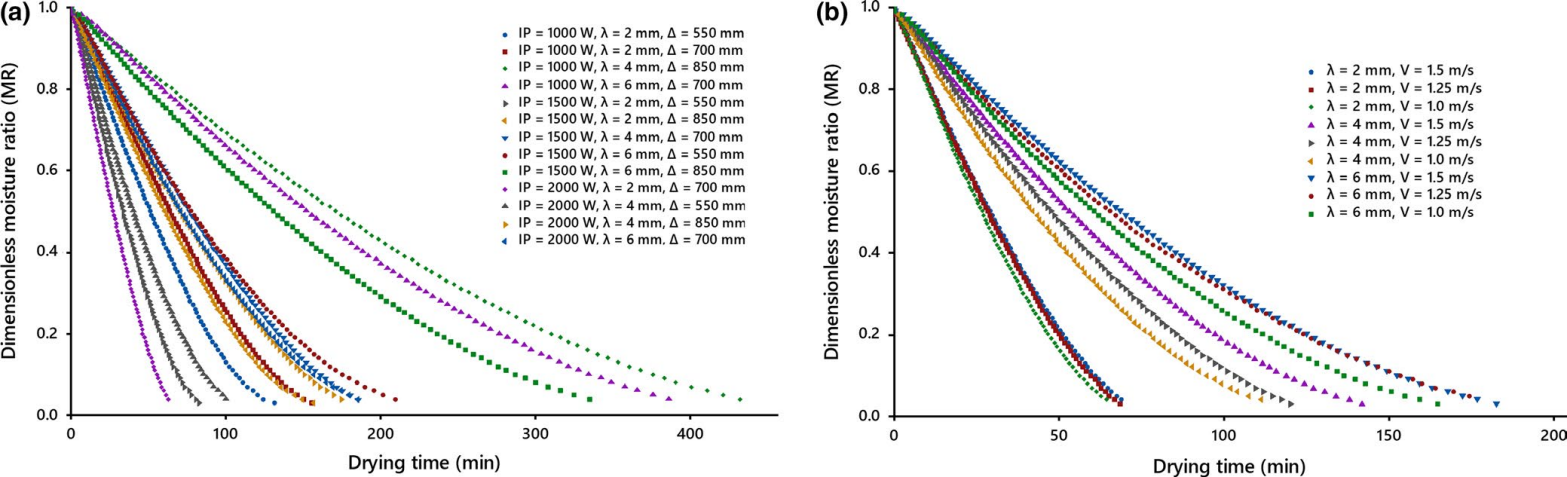
Drying curves of kiwifruit slices at different conditions, (a) under the natural drying air system, (b) at IP = 2,000 W and ∆ = 550 mm under the forced drying air system

Kiwifruit slices with an initial moisture content of about 4.7 d.b. were dried until the final moisture content of 0.2 d.b. in the IR dryer. The product in question is considered as an infinite slab. Drying is interpreted by the decreasing of the moisture ratio of the product, which is the appearance of all the curves of Figure 1.

**Figure 2:**
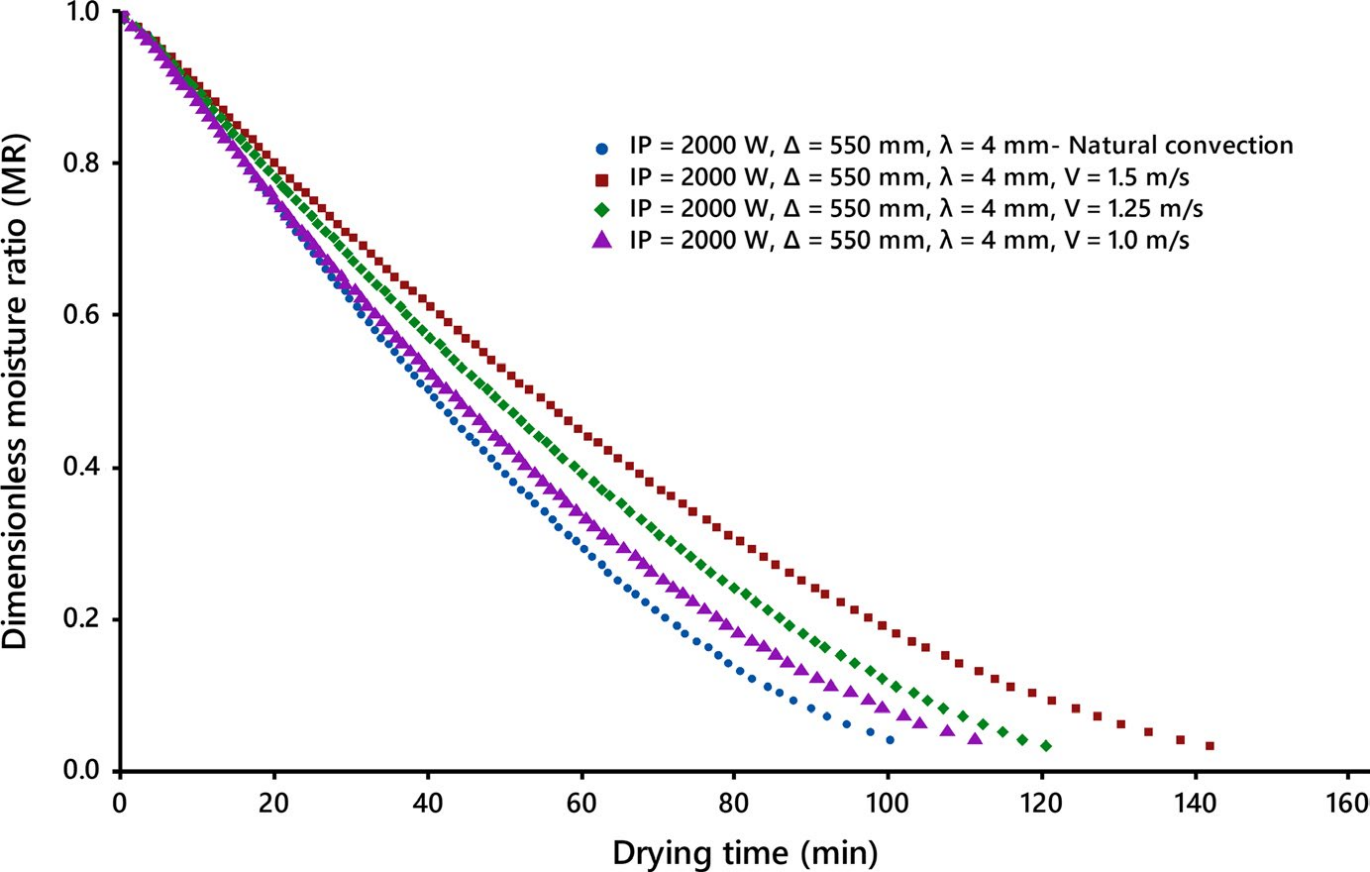
Drying curves of kiwifruit slices at different drying air velocities under the forced drying air system

The studies display an exponential decay and the lack of a constant drying period. In addition, it can be deduced from curves that increasing the slice thickness of the dried product; the distance between slices and infrared lamps; the drying air velocity; and decreasing radiation intensity lead to lengthening of the drying time, as found by Abano, Le Ma, and Qu (2014), Sadin Chegini and Sadin (2014), Sharma, Verma, and Pathare (2005a) and Shi et al. (2008), respectively. The curves reduce rapidly at the beginning and then decrease slowly with increasing drying time, suggesting that diffusion is the most dominant mechanism governing moisture movement in the product under all conditions. The lack of a constant drying rate period can be because of the thin‐layers of the slice that did not provide a constant supply of moisture during drying (Figure 2).

### 3.1 Influence of operating parameters

Figure 1a shows that the radiation intensity is an influential parameter, as was the case in studies presented by Abe and Afzal (1997), Cao et al. (2016), Chayjan et al. (2014), Das et al. (2009), Doymaz (2014a, 2014b, 2014c, 2015b), Kocabiyik and Tezer (2009), Nasiroglu and Kocabiyik (2009), Pathare and Sharma (2006), Wang and Sheng (2006), Wu et al. (2014) and Sharma, Verma, and Pathare (2005b). The considerable effect of the radiation intensity on the drying time can be assigned to that with an increase in the radiation intensity duration drying, the extra energy emanated from IR lamps results in the enhanced surface temperature of slices and drying chamber temperature, leading to an increase in the water vapor pressure and moisture diffusion within the material and its surface, respectively, and finally, results in reducing the drying time.

**Figure 3:**
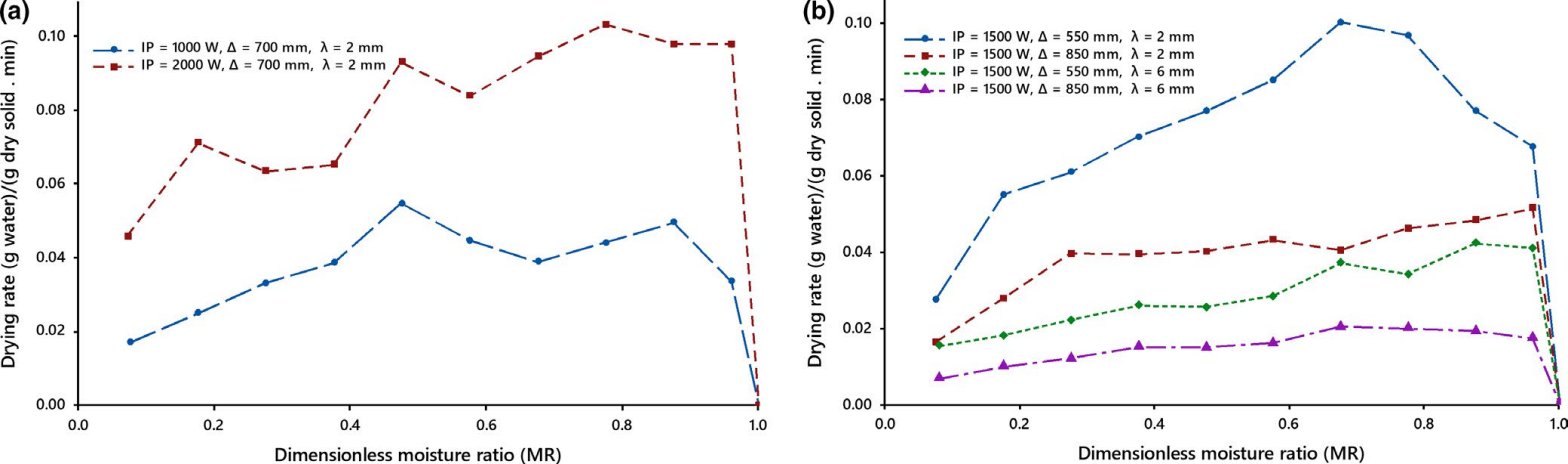
Drying rates of kiwifruit slices, (a) at different IR levels, (b) at different thicknesses and at different distances under the natural drying air system

**Figure 4:**
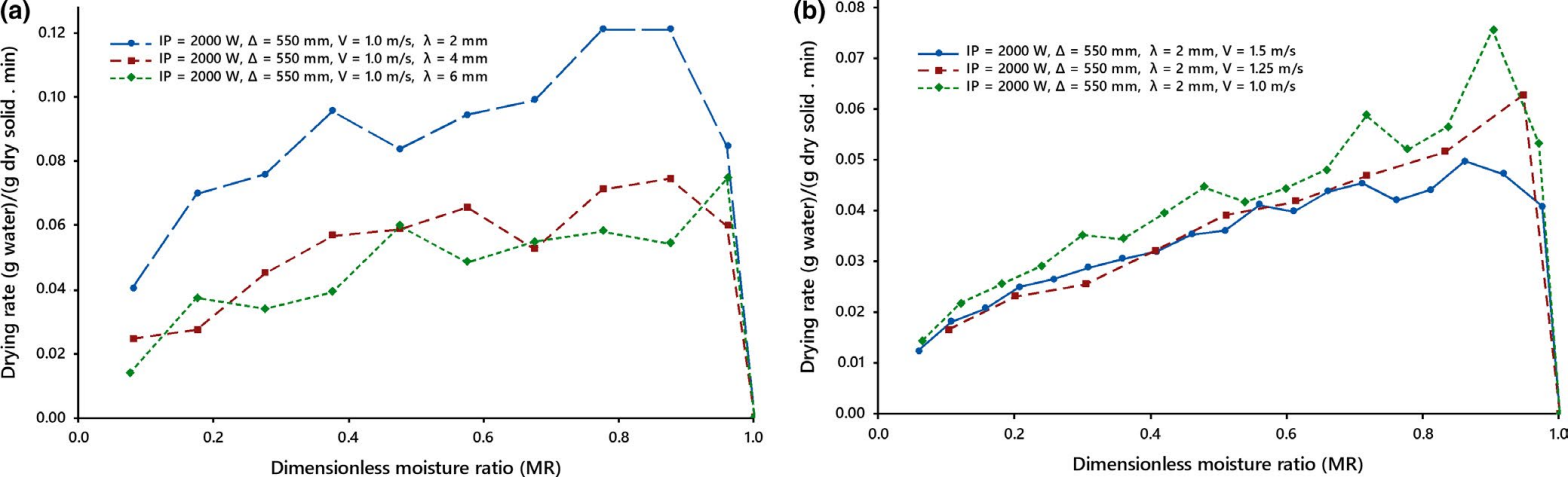
Drying rates of kiwifruit slices, (a) at different thicknesses, (b) at different drying air velocities under the forced drying air system

Figures 3 and 4 show after an initial short period of drying, the drying rate attains a maximum value and then it pursues a falling rate in all drying conditions. Reduction in the moisture ratio leads to a continuous reduction in the drying rate. In the initial stages of drying, the temperature of the kiwifruit slices enhanced sorely owing to absorption of more infrared radiation heat, which indicates a short warming‐up period. It led to the increase of the internal water vapor pressure to enforce the opening of more pores, and thereby a rapid short‐time increase in the drying rates. After this period, drying rates reduced continuously with time under all drying conditions, which demonstrated that the original stage of drying was the falling rate period. Due to the drying of the product surface, heat penetration thru the dried layer reduced thus retarding the drying rates. In addition, the reduction of drying rate might be due to a reduction in porosity of samples due to shrinkage, which enhanced the resistance to moisture movement leading to further fall in drying rates.

During drying stages, the absorption of radiation is affected by the moisture content of the product. Radiation absorption decreases with a reduction in the moisture content of the product, resulting in a lower rate of evaporation, thereby reducing the drying rate.

The average drying rates increased more than two times as infrared intensity level increased from 1,000 to 2,000 W (Figure 3a). These result are in compliance with previous studies on infrared drying of foodstuff (Das et al., 2009; Doymaz, 2014a; Doymaz et al., 2016; Kocabiyik & Tezer, 2009; Sadin et al., 2014; Shi et al., 2008).

The effect of the slice thickness on the drying time can be interpreted as that increasing the exposed surface area resulting in increased diffusion path of moisture out of the slices and following that the increase in the conductive resistance and the moisture gradient of the slice due to the increase of thickness led to an extension in drying time. It would seem that with an increase in the slice thickness, the rate of moisture transfer reduces due to an enhancement of mass transfer resistance (Figures 3b and 4a), thereby leading to the higher moisture content of infrared‐dried kiwifruit slices, as found in previous studies for fruits and vegetables (Abano et al., 2014; Doymaz, 2012; Nowak & Lewicki, 2004; Sharma et al., 2005a).

The significant effect of the distance between slices and infrared lamps on the drying time can be attributed to the fact that the reducing the slice distance from the IR lamps caused to obtain a large amount of heat by material and then resulted in excess enthalpy accumulation within it, which was displayed by an increase in product temperature and finally, led to the reduction of drying times. It was further revealed that as slice thickness increased, the conductive resistance of the slice proliferated, thus leading to reduction of final product temperature. The result is in agreement with previous studies such as infrared drying of tomato slices (Abano et al., 2014) and apple slices (Nowak & Lewicki, 2004).

The drying rates displayed the significant difference with different irradiation distances (Figure 3b), which was similar to result reported by Abano et al. (2014) and Nowak and Lewicki (2004) and was contrary to result reported by Cao et al. (2016). The different results might be owing to the difference in distances and materials used.

As authenticated by the experimental studies performed by Afzal and Abe (1998, 2000), Nowak and Lewicki (2004) and Sharma et al. (2005a), Figures 1b and 2 display that the air velocity is not as influential a parameter as the slice thickness and, in addition, the influence reduces with extension of the drying process. There is no significant change in the positive direction in the drying time for drying air velocity more than 1 m/s (Nowak & Lewicki, 2004). On the contrary, evidence indicates the drying time changes with air velocity.

Increasing the air velocity, as a dissipative parameter, at a given radiation intensity accelerated the cooling effect due to the increase in the mass of air passing through the drying surface, which resulted in lowering of the drying chamber temperature, followed by the slices temperature and moisture vapor pressure, and consequently, the moisture driving force and the drying rate. Its repercussions on the kinetics of drying emerge as the increase in drying time. Researchers such as Afzal and Abe (1998) and Sharma et al. (2005b) have presented similar results. Aghbashlo (2016) reported undesirable loss of the major portion of the absorbed energy without useful application for the moisture removal to the ambient with increasing the air velocity in the infrared drying system.

Similar to scientific findings (Kocabiyik & Tezer, 2009; Nasiroglu & Kocabiyik, 2009; Pathare & Sharma, 2006; Wang & Sheng, 2006), the results suggest that drying rate changes with velocity. It is negatively correlated with air velocity (Figure 4b).

### 3.2 Effective moisture diffusivity

**Figure 5:**
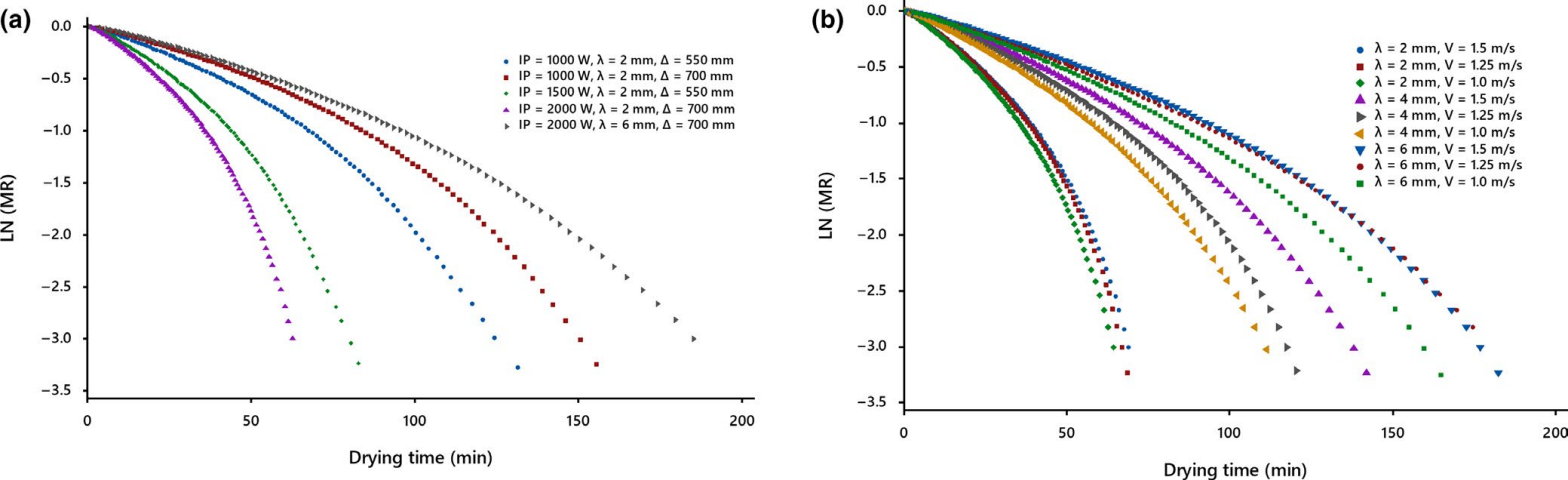
Plots of ln (MR) versus drying time at different conditions, (a) under the natural drying air system, (b) at IP = 2,000 W and ∆ = 550 mm under the forced drying air system

Figure 5 shows a schematic of the changes in the logarithms of moisture ratios against drying time at different drying conditions for both systems. It is clear that the *D*_eff_ enhanced with a reduction in moisture ratio under all drying conditions. It was estimated using Fick's second diffusion (Equation 3). The *D*_eff_ values varied from 1.22–9.0 × 10^−10^ m^2^/s and 2.57–10.33 × 10^−10^ m^2^/s along the experimental range of conditions for natural and forced drying air systems, respectively.

The values of *D*_eff_ are in fact consistent with those in the literature, e.g., 1.17–8.13 × 10^−10^ m^2^/s for infrared drying of sour cherry (Chayjan et al., 2014), 0.73–7.29 × 10^−10^ m^2^/s for infrared drying of carrot slices (Toğrul, 2006), 2.24–16.4 × 10^−10^ m^2^/s for blueberry infrared drying (Shi et al., 2008), 0.62–3.5 × 10^−10^ m^2^/s for onion slices infrared drying (Sharma et al., 2005b), 0.21–5.39 × 10^−10^ m^2^/s for seedless grape infrared drying (Çağlar et al., 2009), 1.31–3.66 × 10^−10^ m^2^/s for sweet potato infrared drying (Doymaz, 2012), 8.04–20.62 × 10^−10^ m^2^/s for mushroom slices infrared drying (Darvishi et al., 2013), 0.72–3.78 × 10^−10^ m^2^/s for watermelon seed infrared drying (Doymaz, 2014a), and 2.38–10.30 × 10^−10^ m^2^/s for catalytic infrared drying of carrot slices (Wu et al., 2014).

The *D*_eff_ values increase with a reduction in distance between the IR lamps and surface of kiwifruit slices, other drying conditions being the same.

When the distance between the IR lamps and the slices is decreased, the temperature of kiwifruit slices is increased and thereby resulting in more evaporation of moisture from the slice surfaces. Similar results were reported for tomato slices (Abano et al., 2014) and onion slices (Sharma et al., 2005b). But, Cao et al. (2016) reported radiation distance shows no significant influence on *D*_eff_.

A two‐factor interaction polynomial relationship and a linear relationship with high *R* ^2^ were found to correlate the effective moisture diffusivity with corresponding operating parameters using DX7 software under the same designs for natural and forced convection systems, respectively, and are given as follows:

(16) $$\begin{array}{c} D_{\text{eff}}=-4.523\times 10^{-10}+3.145\times 10^{-13}\text{IP}+1.102\times 10^{-10}\lambda +6.098\times 10^{-13}\Delta +8.347\times 10^{-14}\text{IP}\lambda -4.655\times 10^{-16}\text{IP}\Delta \\ -1.721\times 10^{-13}\lambda \Delta \;\left(m^{2}/s\right)\;\;\;\;\;R^{2}=0.9974 \end{array}$$

(17) $$D_{\text{eff}}=1.285\times 10^{-10}+1.711\times 10^{-10}\lambda -1.667\times 10^{-10}V\;\;\;\;\;R^{2}=0.987$$

It can be seen from above equations that in the both systems, the slice thickness is more influential than other operating parameters on *D*_eff_ Therefore indicating that the experimental *D*_eff_ values were significantly increased with the slice thickness. For the former system, the highest effective moisture diffusivity derived at IR radiation of 2,000 W, distance of 700 mm between slices, and infrared lamps and slice thickness of 6 mm. It obtained at drying air velocity of 1 m/s and slice thickness of 6 mm for the latter system. It can also be seen from Equation 17 that in the latter system, the air velocity has an effect onhe *D*_eff_ value during infrared drying. Clearly, drying time was prolonged with increasing air velocity and consequently led to further energy consumption (Figure 2).

It was found that the *D*_eff_ enhanced with an increase in IR radiation intensity. This may be because, the increase in radiation intensity led to more energy adsorbed on the sample surface from infrared radiation for moisture evaporation and following a significant variation in the sample internal temperature, which in turn led to the increase in the vapor pressure and consequently led to faster diffusion of moisture toward the surface or the high diffusivity values. The results are similar to the earlier studies of drying red pepper (Cao et al., 2016), blueberry (Shi et al., 2008), sour cherry (Chayjan et al., 2014), and onion slices (Pathare & Sharma, 2006).

The relationship between effective diffusion coefficient, radiation intensity, and activation energy can be given by an Arrhenius equation, other drying conditions being the same.

(18) $$\text{thickness}\;2\;\text{mm}:\;D_{\text{eff}}=4.292\times 10^{-10}\;\exp\left(-\frac{21,376\;m}{P}\right)\;\;R^{2}=0.9963$$

(19) $$\text{thickness}\;4\;\text{mm}:\;D_{\text{eff}}=1.085\times 10^{-9}\;\exp\left(-\frac{16,731\;m}{P}\right)\;\;R^{2}=0.9953$$

(20) $$\text{thickness}\;6\;\text{mm}:\;D_{\text{eff}}=2.303\times 10^{-9}\;\exp\left(-\frac{16,410\;m}{P}\right)\;\;R^{2}=0.9987$$

In the former system, the activation energy for diffusion was calculated to be 21,376, 16,731, and 16,410 W/kg for the thickness of 2, 4, and 6 mm, respectively, and activation energy value at slice thickness of 2 mm was more than other thicknesses. It can also be seen that activation energy decreased for increase in slice thickness during IR drying of kiwifruit. Therefore, the results indicate that the higher effective moisture diffusivities obtained for slices of higher thickness are due to a reduction in activation energy. Afzal and Abe (1998) reported a similar behavior between slice thickness and effective diffusion coefficient and then with activation energy in the product during infrared drying.

The relationship between effective diffusion coefficient, drying medium temperature established by infrared radiation, and activation energy can be given by an Arrhenius equation in the latter system.

(21) $$\text{thickness}\;2\;\text{mm}:\;D_{\text{eff}}=9.31\times 10^{-7}\exp\left(-\frac{2,568.7}{T}\right)\;\;\;\;\;R^{2}≅0.9998$$

The *E*_a_ value of infrared drying of kiwifruit was 21.36 kJ/mol, lower than those of hot air drying (23.6 and 29.6 kJ/mol) reported by Diamante et al. (2010) and Orikasa, Wu, Shiina, and Tagawa (2008), respectively. The *E*_a_ value denotes the sensitivity of moisture diffusivity to temperature, which is related to the structural attributes of the product. So, a higher *E*_a_ value, a greater temperature sensitivity of *D*_eff_. As the infrared radiation increases, the temperature increases as well, resulting in increased energies for the drying process and thus decreases the activation energies. In general, the *E*_a_ values for food and agriculral pructs are in the range 12–130 kJ/mol. (Chayjan et al., 2014).

### 3.3 Modelling of the infrared radiation drying curves of kiwifruit

The moisture content data at the different drying conditions were transformed to the more usable moisture ratio phrase. Results show that drying of kiwifruit slices occurs entirely in the falling rate perd (Figures 1 and 2).

In the present study, 100 models were examined to describe the drying curves of kiwifruit at different conditions (Table 1). It should be noted that some of the models listed in Table 1 were omitted due to issues such as an inadequate fitting with experimental data, failure to implement the model for some experimental data in the software of Minitab 18, the selection of the best mode of a particular model especially in growth models, etc.

The constants of the remaining models were estimated by non‐linear regression technique using software of Minitab 18 for each drying run. A regression analysis was carried out for these models by relating the drying time and dimensionless moisture ratio at different drying conditions with respect to designs proposed by DX7. Then, the operating parameters were correlated with the constants to make the prediction equation more versatile and useful depending on the kind of runs under the same designs. Afterward, the best correlation was selected with testing different transformations for obtaining the highest value of determination coefficient using DX7. After replacing the correlated equations of constants in the models, the predicted values of each model were ultimately compared with the experimental ones.

Goodness of fit of the models is characterized by the higher values of *R* ^2^ and EF and lower values of *χ* ^2^, RMSE, *P*, and MBE. The statistical results of the different models, comprising the criteria mentioned above to evaluate goodness of fit were presented in Table 2.

**Table 2:** Statistical analysis of models at different operating conditions

Model no.	*R* ^2^		RMSE		*χ* ^2^		*P*			EF		MBE	
(a) Under natural drying air convection													
2	0.834722159		0.112966961		0.01276447		23.7153287			0.83365726		0.022233	
4	0.977223539		0.041811949		0.00174864		11.4659595			0.97721223		0.006172	
6	0.978585108		0.040536119		0.00164374		10.6038209			0.97858169		0.003502	
7	0.834722159		0.112966961		0.01276447		23.7153287			0.83365726		0.022233	
8	0.991658663		0.025297038		0.00064016		7.78173215			0.99165857		0.000906	
9	0.97992744		0.039270125		0.00154267		11.1461678			0.97989863		0.010493	
13	0.996481095		0.016430607		0.00027006		5.85139977			0.99648109		−0.00035	
14	0.952220411		0.060552282		0.00366826		14.675159			0.95220725		0.004597	
15	0.983573195		0.03550092		0.00126075		8.68174345			0.98357217		0.002188	
16	0.967650942		0.049819381		0.00248254		15.5166419			0.96764826		−0.00252	
17	0.973318899		0.045243467		0.00204768		13.5289887			0.97331835		0.001259	
27	0.957269117		0.057264878		0.00328191		13.0215342			0.95725576		0.004898	
30	0.948064897		0.063122986		0.00398543		18.5082662			0.9480631		−0.00163	
31	0.995795638		0.017959721		0.00032274		5.91767972			0.99579564		3.63861E‐05	
33	0.995872363		0.017796064		0.00031677		6.66540721			0.99587191		0.002892595	
36	0.97590571		0.042997121		0.00184918		10.867135			0.97590207		0.003404	
37	0.998082204		0.012129738		0.0001472		3.5924019			0.9980822		0.000494	
41	0.997360738		0.014208263		0.00020194		4.61896929			0.99737255		−0.00039	
44	0.982623371		0.036511804		0.00133372		10.1499036			0.98262329		0.000598	
45	0.954178233		0.059290836		0.00351662		12.7253158			0.95417778		0.00087	
46	0.988701377		0.029441673		0.00086721		9.09176696			0.98870137		0.000276	
47	0.9964061		0.016604916		0.00027588		4.471826			0.99640603		0.001215	
48	0.987967628		0.030383188		0.00092356		10.6045531			0.98796717		0.001702	
51	0.976752159		0.042232462		0.0017842		11.8308259			0.97675156		0.001402	
53	0.995498621		0.018583359		0.00034546		6.1894179			0.99549858		0.000823	
55	0.997896551		0.012703272		0.00016141		4.41609498			0.99789655		0.000216	
59	0.996662846		0.01600066		0.00025611		5.66302271			0.99666284		0.00028	
62	0.981341486		0.037777991		0.00142783		10.6350374			0.98142497		0.000589	
65	0.997139374		0.014792111		0.00021891		5.1158652			0.99715218		0.000307	
69	0.998228015		0.011642066		0.0001356		3.9959008			0.99823595		−0.00048	
81	0.998247792		0.011576903		0.00013407		3.97323707			0.99825564		−0.00029	
86	0.998430807		0.010955669		0.0001201		3.4412919			0.99843782		−0.00069	
87	0.998722584		0.009899555		9.8035E‐05		3.63627378			0.99872258		3.03E‐05	
88	0.946930922		0.063807293		0.00407324		10.8386969			0.94693091		−0.00012	
93	0.995853096		0.017810871		0.0003173		6.67554146			0.99587122		0.002895	
95	0.996693972		0.015902051		0.00025293		5.63553901			0.99670877		0.000424	
97	0.998327552		0.011310358		0.00012798		3.68432174			0.99833504		−0.00048	
	**Feed‐forward neural networks**												
100	**Topology**	**Transfer functions**		***R*^2^**		**RMSE**		***P***	**EF**		**MBE**		**Computing time (s)**
	4‐7‐1	TANSIG		0.999614974		0.005434939		1.78264526	0.99961497		4.01453E‐05		30
	4‐7‐1	LOGSIG		0.999481526		0.00630686		2.2086185	0.99948153		3.75954E‐05		21
	4‐9‐1	TANSIG		0.999893979		0.002851969		0.85250294	0.99989398		−2.8327E‐05		173
	4‐9‐1	LOGSIG		0.999871785		0.003136306		0.86107002	0.99987179		−0.000116308		66
	4‐3‐3‐1	LOGSIG ‐LOGSIG		0.999423115		0.006652641		2.24044421	0.99942312		−1.9216E‐05		123
	4‐3‐5‐1	TANSIG‐ TANSIG		0.999469121		0.006381861		2.01444074	0.99946912		−8.91392E‐05		95
	4‐3‐5‐1	LOGSIG ‐LOGSIG		0.999361254		0.007000251		2.13763784	0.99936125		−2.43911E‐05		207
	4‐5‐5‐1	TANSIG‐ TANSIG		0.999945895		0.00203736		0.58540218	0.9999459		1.75347E‐05		169
	4‐5‐5‐1	LOGSIG ‐LOGSIG		0.999959194		0.001769346		0.35594154	0.99995919		1.15015E‐05		280
	4‐5‐7‐1	TANSIG‐ TANSIG		0.999975057		0.001383333		0.27730406	0.99997506		−1.6075E‐05		68
	4‐5‐7‐1	LOGSIG ‐LOGSIG		0.999956769		0.001821157		0.50997122	0.99995677		−2.98673E‐05		87
	4‐7‐7‐1	TANSIG‐ TANSIG		0.999993783		0.000690635		0.1844771	0.99999378		−4.73051E‐06		325
	4‐7‐7‐1	LOGSIG ‐LOGSIG		0.999980004		0.001238567		0.26836835	0.99998		−1.44851E‐05		334
	4‐7‐9‐1	TANSIG‐ TANSIG		0.999988534		0.00093789		0.17848912	0.99998853		5.13478E‐06		337
	4‐7‐9‐1	LOGSIG ‐LOGSIG		0.999990807		0.000839826		0.15818268	0.99999081		−2.06303E‐06		260
	4‐9‐9‐1	TANSIG‐ TANSIG		0.999993675		0.000696608		0.14657346	0.99999367		−8.87648E‐07		277
	4‐9‐9‐1	LOGSIG ‐LOGSIG		0.999994617		0.000642626		0.14215229	0.99999462		6.63169E‐07		268
	4‐8‐14‐1	TANSIG‐ TANSIG		0.999997161		0.000466686		0.11846808	0.99999716		5.73108E‐06		405
	4‐18‐18‐1	TANSIG‐ TANSIG		0.99999744		0.000443186		0.11259975	0.99999744		1.30963E‐06		1,494

Model no.	*R* ^2^		RMSE		*χ* ^2^		*P*			EF		MBE	
(b) Under forced drying air convection													
2	0.996137814		0.017242319		0.00029742		6.85526255			0.99613731		0.003176	
4	0.995916739		0.017728984		0.00031445		6.97726217			0.99591618		0.003243	
6	0.996784377		0.015732017		0.00024765		5.87319695			0.99678436		0.000659	
8	0.992916052		0.023351167		0.00054562		8.21206933			0.99291539		0.002682	
9	0.991487286		0.025597318		0.00065563		8.72928198			0.9914869		0.001866	
13	0.997995486		0.012421044		0.00015438		4.30300063			0.99799546		−0.00102	
14	0.998713486		0.009950813		9.9101E‐05		3.50734397			0.99871348		−0.00051	
15	0.998667329		0.010127748		0.00010264		3.56790666			0.99866732		−0.00052	
16	0.996450197		0.016530663		0.00027338		5.77458156			0.99644958		−0.00365	
17	0.997809326		0.012984943		0.00016871		4.58822132			0.99780932		−0.00044	
20	0.994813348		0.019981028		0.00039941		8.48062599			0.99481278		0.002895	
24	0.999068066		0.008469216		7.1832E‐05		2.89364332			0.99906807		−0.0001	
27	0.999068066		0.008469216		7.1832E‐05		2.89364332			0.99906807		−0.0001	
30	0.996645604		0.016068737		0.00025831		5.5899145			0.99664523		−0.00292	
33	0.995606421		0.018390543		0.00033835		7.15112635			0.99560572		0.003509062	
36	0.998115449		0.012043551		0.00014511		4.29688937			0.99811545		0.000122	
37	0.998530329		0.010635618		0.00011321		3.70889555			0.99853032		−0.00086	
41	0.99762811		0.013486025		0.00018199		4.88109186			0.99764141		8.8E‐05	
43	0.983714518		0.035403889		0.00125448		12.5001133			0.98371451		−0.00014	
44	0.998397031		0.01110742		0.00012348		3.80232209			0.99839703		9.77E‐05	
46	0.998702042		0.009994976		9.9983E‐05		3.52013941			0.99870204		−0.00052	
47	0.997995422		0.012421244		0.00015445		4.30312996			0.99799539		−0.00103	
48	0.988252757		0.030101779		0.00090687		9.70453438			0.98822711		−0.01296	
51	0.996520949		0.016363681		0.00026794		5.78989808			0.99652095		2.75E‐06	
53	0.997235848		0.014586163		0.00021289		5.57574491			0.99723573		0.001832	
55	0.998422821		0.011017716		0.00012144		4.1579513			0.99842282		0.000427	
56	0.995538321		0.044225288		0.0019571		18.2778302			0.99553832		0.000329	
58	0.998439295		0.010960178		0.00012018		3.53686988			0.99843925		−0.00153	
59	0.997040868		0.015091553		0.0002279		5.70881086			0.99704085		0.000641	
65	0.997821168		0.012925542		0.00016721		4.58471377			0.99783338		0.000237	
69	0.997799001		0.012991122		0.00016891		4.65666417			0.99781134		9.76E‐05	
81	0.998169917		0.01184604		0.00014042		4.27693076			0.99818017		0.000619	
86	0.998233707		0.011638073		0.00013559		4.39189496			0.99824351		0.002146	
87	0.997995512		0.012420965		0.00015438		4.30309192			0.99799548		−0.00103	
93	0.995714108		0.018129661		0.00032882		7.10692923			0.9957375		0.003382	
95	0.996592543		0.016164193		0.00026139		6.03601235			0.99661161		0.000877	
97	0.997506654		0.013827001		0.00019135		4.86636031			0.99752063		−7.32656E‐05	
100	**Feed‐forward neural networks**												
	**Topology**	**Transfer functions**		***R*^2^**		**RMSE**		***P***	**EF**		**MBE**		**Computing time (s)**
	3‐3‐1	LOGSIG		0.999103098		0.008308508		2.84055304	0.9991031		−6.31998E‐05		23
	3‐5‐1	TANSIG		0.999697456		0.004825527		1.43846276	0.99969746		5.3265E‐05		53
	3‐5‐1	LOGSIG		0.999467193		0.006403768		2.21694224	0.99946719		1.11919E‐05		7
	3‐7‐1	TANSIG		0.999924775		0.002406197		0.77379453	0.99992478		2.02833E‐06		19
	3‐7‐1	LOGSIG		0.999819958		0.003722522		1.25101896	0.99981996		2.65846E‐05		45
	3‐9‐1	TANSIG		0.999969821		0.001524066		0.3221763	0.99996982		−4.94208E‐06		120
	3‐9‐1	LOGSIG		0.99996917		0.001540415		0.31350872	0.99996917		−9.08951E‐06		86
	3‐2‐3‐1	TANSIG‐ TANSIG		0.999320388		0.007232376		2.46546678	0.99932039		6.79236E‐05		47
	3‐2‐3‐1	LOGSIG ‐LOGSIG		0.999434439		0.006597666		2.3345221	0.99943444		7.3034E‐06		164
	3‐3‐3‐1	TANSIG‐ TANSIG		0.999645872		0.005220726		1.85014582	0.99964587		−6.25248E‐05		25
	3‐3‐3‐1	LOGSIG ‐LOGSIG		0.999826081		0.003658674		1.14242859	0.99982608		3.90497E‐05		85
	3‐3‐5‐1	TANSIG‐ TANSIG		0.99994893		0.001982598		0.48338989	0.99994893		−3.32468E‐05		61
	3‐3‐5‐1	LOGSIG ‐LOGSIG		0.999437087		0.006582201		2.01095337	0.99943709		−8.48547E‐05		128
	3‐5‐5‐1	TANSIG‐ TANSIG		0.999949299		0.001975423		0.40947451	0.9999493		−6.71082E‐07		27
	3‐5‐5‐1	LOGSIG ‐LOGSIG		0.999983617		0.001122901		0.2741601	0.99998362		5.69948E‐06		52
	3‐5‐7‐1	TANSIG‐ TANSIG		0.999989388		0.000903748		0.2377551	0.99998939		5.39138E‐06		92
	3‐5‐7‐1	LOGSIG ‐LOGSIG		0.999975134		0.001383411		0.25558603	0.99997513		−2.35667E‐06		177

Among the empirical, semitheoretical and the growth curve models, model 87 gave a highest *R* ^2^, EF values and lowest RMSE, *χ* ^2^, and MBE and thus, was selected to represent the infrared drying of kiwifruit for the natural drying air system. In the same manner, models 24 or 27 were selected for the forced drying air system. Because the statistical results of the two models are the same. In other words, model 24 and model 27 are statistically similar. In this case, it seems that model 27 is chopped form of model 24. model 87 and model 24 are given as follows:

$$\text{Model}\;\text{exponential}\;\text{dacay}\;\text{function}\left(87\right):\;\text{MR}=c+\left(d-c\right)\exp\left(-t/e\right)$$

$$\begin{array}{c} 1/c=-7.7959+9.61\times 10^{-4}\text{IP}-0.50755\lambda +1.96\times 10^{-2}\Delta -8.7\times 10^{-5}\text{IP}\lambda +2.6\times 10^{-7}\text{IP}\Delta +7.36\times 10^{-4}\lambda \Delta -2.5\times 10^{-7}{\text{IP}}^{2}\\ -0.01417\lambda^{2}-1.6\times 10^{-5}\Delta^{2} \end{array}$$

$$\begin{array}{c} d=0.962504+1.39\times 10^{-5}\text{IP}+0.006396\lambda +1.72\times 10^{-4}\Delta +4.29\times 10^{-6}\text{IP}\lambda -1.5\times 10^{-9}\text{IP}\Delta -9.5\times 10^{-6}\lambda \Delta -7.9\times 10^{-9}{\text{IP}}^{2}\\ -0.00072\lambda^{2}-1.2\times 10^{-7}\Delta^{2} \end{array}$$

$$\begin{array}{c} 1/\sqrt{e}=1.296293+3.47\times 10^{-4}\text{IP}-0.13414\lambda -1.79\times 10^{-3}\Delta -1.5\times 10^{-5}\text{IP}\lambda -2.2\times 10^{-7}\text{IP}\Delta +1.68\times 10^{-5}\lambda \Delta +3.43\times 10^{-8}{\text{IP}}^{2}\\ +0.012579\lambda^{2}+1.04\times 10^{-6}\Delta^{2} \end{array}$$

$$\text{Model}\;\text{modified}\;\text{two - term}\;\text{exponential}-V\left(24\right):\;\text{MR}=a\;\exp\left(-k_{0}t^{n}\right)+b\;\exp\left(-k_{1}t\right)$$

$$a=1.146-2.54\times 10^{-2}\lambda -0.84833V+1.95\times 10^{-2}\lambda V-0.0011\lambda^{2}+0.30144V^{2}$$

$$k_{0}=6.599259-1.663\lambda -2.61162V+0.146\lambda V+0.134002\lambda^{2}+0.620853V^{2}$$

$$n=0.869619-0.0911\lambda +2.40274V-0.0519\lambda V+0.013111\lambda^{2}-0.81963V^{2}$$

$$b=-0.1297+0.025456\lambda +0.853694V-0.0202\lambda V+0.001342\lambda^{2}-0.30192V^{2}$$

$$\begin{array}{c} k_{1}=7.871613-1.4202\lambda -3.20975V+0.10178\lambda V+0.103414\lambda^{2}\\ +0.88928V^{2} \end{array}$$

Model 100 in Table 2 summarizes the list of the best neural networks published in the literature and as well suggested topologies. The total data obtained for natural and forced systems are 8,705 and 4,806, respectively. In order to compare the empirical, semitheoretical and the growth curve models with neural networks, LM training algithm has been used for all of them. It can be seen from the statistical analysis (Table 2) that the all topologies presented in Table 2 provide better results toward the empirical, semitheoretical and the growth curve models.

In general, computing time and accuracy increase with an increase in the number of hidden layers and neurons. In addition, their values alter with the change of transfer functions. If statistical analysis is the only criterion of the assessment of the process, the topologies of 4‐18‐18‐1 and 3‐5‐7‐1 with the LOGSIG ‐LOGSIG transfer functions are the best choices for natural and forced drying air systems, respectively. If the computing time and simplicity of the topology are considered, the best choices are the topology of 3‐7‐1 with the TANSIG transfer function and the topology of 4‐7‐1 with the LOGSIG transfer function for ANNs with one hidden layer for the former and latter systems, respectively. If attention is focused on more accuracy, computing time and analytical simplicity simultaneously, the topologies of 4‐5‐7‐1 and 3‐5‐5‐1 with the TANSIG‐ TANSIG transfer functions for ANNs with two hidden layers for natural and forced drying air systems, respectively, are the best options to predict the infrared drying curves of kiwifruit.

**Figure 6:**
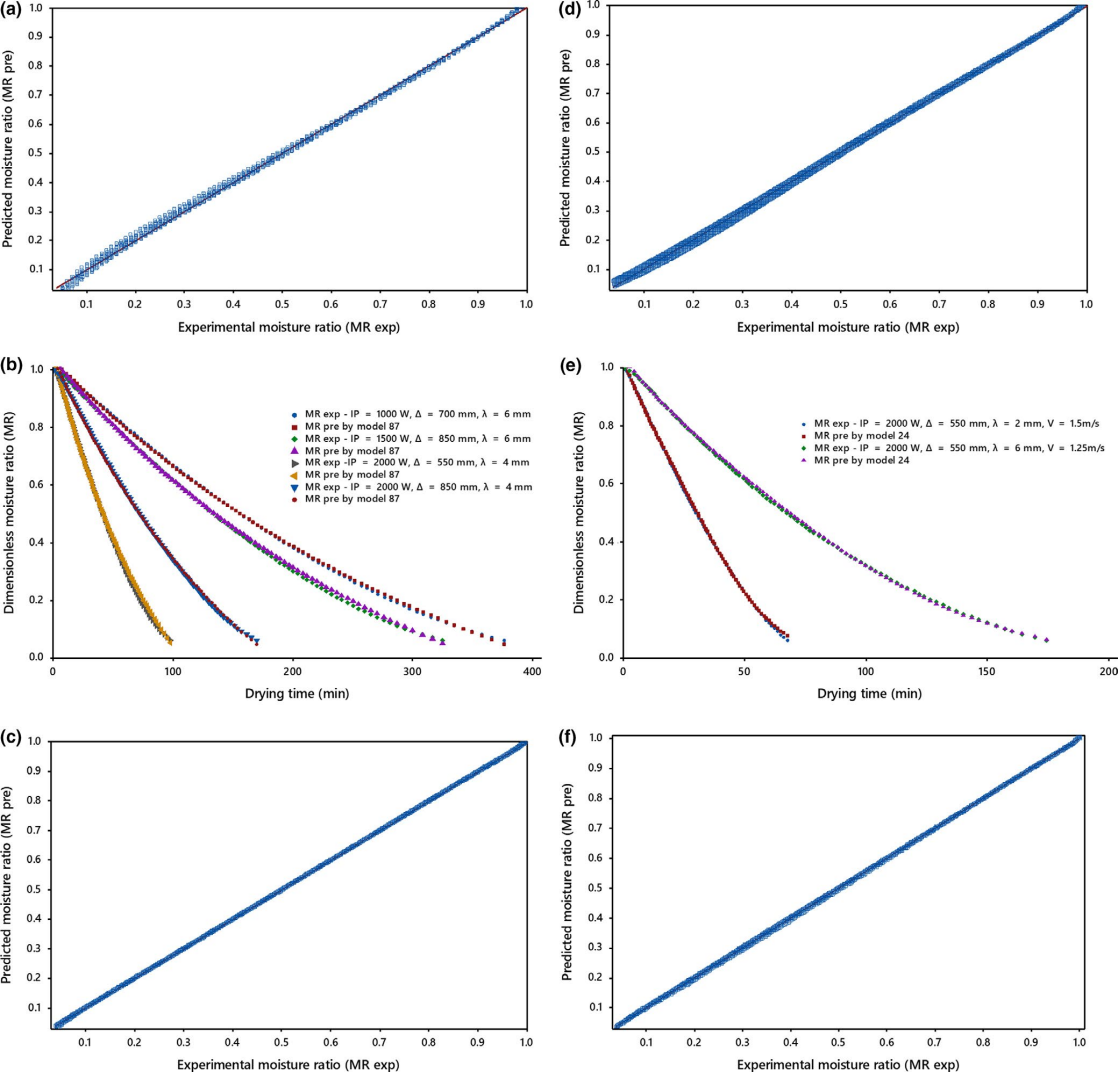
(a) comparison of the experimental and predicted moisture ratio values from model 87, (b) variation of experimental and predicted moisture ratio with drying time for the selected model, (c) predicted values of moisture ratio using ANN with topology of 4‐5‐7‐1 versus experimental values under the natural drying air system and, (d) comparison of the experimental and predicted moisture ratio values from model 24, (e) variation of experimental and predicted moisture ratio with drying time for the selected model, (f) predicted values of moisture ratio using ANN with topology of 3‐5‐5‐1 versus experimental values under the forced drying air system

Experimental data were compared with those predicted by the model 87 and model 24 for relevant systems in Figure 6. The predictions using the model 87 and model 24 displayed MR values banded around a 45° straight line on the plots for natural and forced drying air systems, respectively, which displayed the suitability of these models to describe the infrared drying behavior of kiwifruit under various conditions. In addition, experimental data were compared with those predicted by the ANNs with the topologies of 4‐5‐7‐1 and 3‐5‐5‐1 for relevant systems in the figure. The comparison between models 24, 87 and the best ANNs showed that ANNs modelling could be effectively used for prediction of infrared drying curves.

## 4 CONCLUSION

Kiwifruit drying behavior in a laboratory infrared dryer at three levels of radiation intensity, slice thickness, and distance between slices and infrared lamps under natural drying air system, at three levels of slice thickness and air velocity under forced drying air system was studied. It was dependent on the radiation intensity, the slice thickness, the distance between slices and infrared lamps, and the drying air velocity; i.e., the drying time decreased with increasing the radiation intensity and decreasing the slice thickness, the distance between slices and infrared lamps, and the drying air velocity. The falling drying rate period was only observed during infrared drying of kiwifruit slices in all runs for both systems.

The effective moisture diffusivities of the kiwifruit slices increased and correlated to a polynomial relationship with decreasing distance between infrared lamps and surface of slices and increasing radiation intensity and slice thickness for natural drying air system. In addition, they increased and correlated to a linear relationship with decreasing drying air velocity and increasing slice thickness for forced drying air system.

Slice thickness was found to be dominant for internal moisture movement during IR drying of kiwifruit for both systems. The effective moisture diffusivities ranged between 1.22–9.0 × 10^−10^ m^2^⁄s and 2.57–10.34 × 10^−10^ m^2^⁄s for natural and forced drying air systems, respectively, and were in agreement with values reported in the literature for IR drying of foodstuff.

For the former system, activation energies of 21.376, 16.731, and 16.41 kW/kg were obtained and were inversely proportional to the thickness of slices, and for the latter system, an activation energy of 21.36 kJ/mol was obtained by an Arrhenius equation for the slice thickness of 2 mm.

Among the empirical, semitheoretical and the growth curve models fitted to the infrared drying data, model 87 and model 24 gave the best fit for the former and latter systems, respectively. The ANNs with topologies of 4‐5‐7‐1 and 3‐5‐5‐1, TANSIG transfer function and the LM training algorithm were found to be the best for prediction of variations in the kiwifruit moisture ratios during infrared drying for natural and forced drying air systems, respectively.

## CONFLICT OF INTEREST

The authors declare that they have no conflict of interest.

## ETHICAL STATEMENT

This study does not involve any human or animal testing.

## INFORMED CONSENT

For this type of study, formal consent is not required.
